# Exploring the benefits, facilitators and barriers to male involvement in sexual and reproductive health: a qualitative study of nurses’ and midwives’ perspectives in Ghana

**DOI:** 10.3389/frph.2026.1847732

**Published:** 2026-07-13

**Authors:** Evelyn Asamoah Ampofo, Caroline Dinam Badzi, Berit Mortensen

**Affiliations:** 1 School of Nursing and Midwifery, University of Ghana, Accra, Ghana; 2 Faculty of Health Sciences, Department of Nursing and Health Promotion, OsloMet - Storbyuniversitetet, Oslo, Norway

**Keywords:** benefits, facilitators, Ghana, male involvement, midwives & nurses, reproductive health, sexual and reproductive health

## Abstract

**Background:**

Male involvement in sexual and reproductive health (SRH) though a relatively old concept in Ghana continues to evolve traditionally focusing on women’s health. The World Health Organization has encouraged gender-inclusive maternal health services to leverage men’s roles in supporting their partners’ health.

**Aim:**

This study aims to explore the perspectives of nurses and midwives on male involvement in SRH, identifying benefits, challenges, and strategies for enhancement.

**Method:**

A qualitative exploratory design was employed in Greater Accra Region, Ghana, engaging 32 nurses and midwives through five focus group discussions. Data collection involved using a semi-structured interview guide and recorded interviews. Data analysis was based on Braun and Clark’s thematic analysis framework.

**Results:**

Findings revealed three primary themes: benefits of male involvement, barriers to participation, and strategies to enhance engagement. Participants noted significant positive outcomes, including improved health outcomes for women, reduced complications due to emotional and logistical support by male partners. However, barriers such as cultural norms, societal expectations and inadequate knowledge hindered active male involvement. This notwithstanding, participants have a strong desire to promote male involvement.

**Discussion:**

Results underscore the necessity of comprehensive education and community engagement to reshape attitudes towards male involvement. The lack of formal training for healthcare providers on facilitating male participation was identified as a critical gap.

**Conclusion:**

Enhancing male involvement in SRH can significantly improve maternal health outcomes. Nurses and midwives play a pivotal role in promoting this initiative through targeted education, outreach programs, and policy advocacy, ultimately fostering healthier family dynamics in Ghanaian society.

## Background

The push towards the implementation of male involvement in Sexual and Reproductive Health, is relatively new in Ghana, however, the concept has existed for over two decades and continues to evolve. Traditionally, issues concerning reproductive health have focused exclusively on women. As far back as 2001, the World Health Organization proposed recommendations to extend the scope of maternal health services to include men in the reproductive health of their partners (1). This decision was informed by the recognition that men have a unique role to play in promoting safe maternal health and should not be viewed as passive onlookers. Several strategies have subsequently been suggested to promote male involvement; however, a lot more needs to be done to maximise the full benefits. These strategies include but not limited to encouraging male partner participation in maternal and child health (MCH) services, creating community awareness and sensitization, engaging men in MCH departments, providing additional health services that address men's health needs, and reducing waiting time for women who come to seek services with male partners (2).

Although male involvement is recognised, most of the programmes and services have been designed to target women during antenatal, labour, and postpartum periods, and in the provision of comprehensive abortion care and family planning services. Where male involvement is acknowledged, the level of involvement varies depending on some individual and contextual factors, including sociodemographic factors such as the partner's education, type of marriage arrangement and the number of children to mention a few. In addition to these, distance to health facilities, cultural norms, gender roles and health worker attitudes have determined the level of male involvement (3, 4).

The definition of male involvement varies depending on the aspect of care being considered at a particular point and the context in which care occurs. Male involvement in reproductive, maternal, and newborn services is a practice wherein fathers and male community members actively participate in caring for and supporting the family to access better health services (5). Male involvement describes the process of social and behavioural change needed for men to play more responsible roles in maternal health care to ensure that women and children enjoy good health.

In the Ghanaian context, male involvement in maternal healthcare is conceptualised as the involvement of men or the clinical attendance of men with their spouses and participation of men in the traditionally assigned female gender roles such as household chores (cooking, cleaning fetching water) especially at a time when a woman is pregnant or has given birth (6). Even though the role of men is key and strongly recognised in the family system in Ghana, male involvement in sexual and reproductive health (SRH) cannot be assumed to be automatic. The Ghana Health Service officially recognises the importance of male involvement and, as part of its strategic plan over the years, has emphasised male involvement in maternal health services as an essential component of improving maternal health outcomes (7).

The benefits of male involvement in SRH are well documented in the literature (4, 8). There is a need to emphasise that men could be greatly affected by the social, cultural and economic influence of improved or adverse maternal health outcomes. As such, they (men) are to be adequately informed and involved in sexual and reproductive health interventions. Because men are adversely affected by the morbidity and mortality of their wives and female relatives (9), they need support from healthcare providers to recognise and understand the factors contributing to maternal mortality and morbidity.

The effective inclusion of men in SRH initiatives in Ghana provides a promising avenue for enhancing maternal health outcomes. By actively engaging male partners in reproductive health discussions and services, healthcare providers can foster healthier family dynamics that benefit both women and their male counterparts, ultimately contributing to a reduction in maternal mortality and the improvement of overall family health.

Despite the numerous benefits of male involvement there are some barriers that have been identified through several studies (10, 11). These barriers include sociocultural norms, gendered roles, and a lack of knowledge about reproductive and maternal health (5). Moreover, most studies on male involvement have considered male participants. It is important to involve the healthcare providers who interacts with both the women receiving care and the supporting partners on barriers and facilitators for male involvement (12, 13). Although several studies have been conducted on male involvement, majority investigated the phenomenon from the perspective of males and female clients/patients. The perspective of healthcare providers who are primary implementers and promoters of this strategy has not received the needed attention. Male involvement beyond its socioeconomic role towards women accessing reproductive health services is explored in this study. This study defines male involvement as physical presence of male partners, especially during hospital attendance, their participation in decision making in addition to the economic support.

This study engaged nurses and midwives who provide SRH services to explore their perspectives on male involvement. The aim was to understand the benefits, challenges and ways of enhancing the concept of male involvement in SRH so that strategies to upscale benefits can be considered to enhance the well-being of families.

## Methods

### Study design

The study adopted a qualitative research approach, specifically the exploratory- descriptive qualitative design (EDQ). This design was used to conduct the study in the Greater Accra Region in Ghana. The EDQ design is appropriate for investigating an issue or problem that needs a solution or understanding (14). It is often suitable to studying areas within health care that have previously received little or no attention, with the aim of exploring and describing the phenomenon of interest (15). The design is suitable for this study which sought to explore the perceived benefits, facilitators and barriers from the perspectives of nurses and midwives since research on male involvement from their perspective is limited. The design allowed participants to freely express themselves and share their experiences on engaging males in SRH.

### Study setting

The setting for the study was the Greater Accra Region. Service delivery within the health system in Ghana is organised as a five-tiered system made up of the National, Regional, District, Sub-district, and Community Health Planning and Services. There are other quasi-governmental health service providers, such as the security services and faith-based facilities (7). The Greater Accra Region has several facilities that represent the five-tiered system. All the different levels of service provide reproductive health services, including antenatal, labour, postnatal, and family planning services. This made it possible to recruit participants from each level.

### Participants and recruitment

Following ethical clearance by the Ghana Health Service Ethics Review Committee (GES-ERC:006/10/22), heads of eleven (11) selected health facilities within Greater Accra were contacted by the research team to help identify nurses and midwives directly involved in the delivery of SRH services. These facilities were mostly secondary levels with a few tertiary level ones. It is significant to note that in Ghana nursing and midwifery is a female dominated profession. Midwifery training institutions in Ghana generally do not enrol males. Additionally, most SRH services are provided by midwives. It is therefore not surprising that only one male nurse was available and willing to participate in the study. Thirty-two nurses and midwives were purposively selected and recruited after the details of the study had been explained to them. The number of participants for each facility varied depending on the availability of qualified individuals who met the inclusion criteria. Five (5) focus groups made up of a minimum of 6 and a maximum of seven participants in each group were created. There was no need to recruit more participants as data saturation was reached with the five focus group discussions made up of a total of 32 participants. Each focus group had a facilitator who assigned each participant a code.

The five focus groups were identified with the alphabets A, B, C, D and E. Participants in each group were randomly assigned numbers from one to six or seven depending on the number of participants in a group. Hence participants had codes such as A1, A2 or B1 B3 etc. depending on the group and the assigned number. The letter represented the focus group (A-E) while the number represents the participant within that group (e.g., A1, A2) Hence participants had codes such as A1, A2 or B1 B3 etc. depending on the group and the assigned number within their respective groups. The purpose of the code was to facilitate the discussion among participants without using their real names or identities. Before the discussion began, the facilitators reassured the participants of confidentiality and encouraged them to relax. Icebreakers were used to help participants feel relaxed, while confidentiality was ensured by using no participant identifiers.

### Inclusion and exclusion criteria

Nurses and midwives in selected facilities who had a minimum of one year's service provision for reproductive health services were recruited for the study. Those who had less than a year's experience or were not available were excluded.

### Data collection

Data collection took place at the School of Nursing and Midwifery of the University of Ghana. Prior to data collection, the focus group discussion guide was pretested among nurses and midwives from the University of Ghana School of Nursing and Midwifery. Afterward, the facilitators for the focus group discussions met, discussed, and agreed on the revised focus group discussion guide. The guide was organised into four sections. The first section had questions on the definition of SRH and maternal health in general. The second section had questions on how these services were provided in their respective health facilities. Questions included family planning, comprehensive abortion care, ante- and postnatal services, prevention and management of sexually transmitted infections, male involvement in SRH and, maternal health services. The third section focused on identifying the challenges or barriers faced in providing these services. The fourth section aimed to gather insights on what midwives and nurses require to enhance the delivery of these services.

On the day of data collection, the research team welcomed the participants, explained, the purpose of the study, and gave them the opportunity to ask questions. Each participant was privately taken through the informed consent process to sign a written consent form after which they were randomly assigned to the five focus groups.

Before starting the discussions, participants were asked for their consent to audio record the sessions solely for research purposes. The participants’ permission was sought to audio record the interactions purely for research purposes. A qualitative expert transcribed the interviews using standardised procedures to ensure the anonymity of all transcripts. The PI managed and stored all study data, audio files, and notes taken during the focus group discussions on a password-protected computer. Data collection was conducted in English. Ethical approval for the overall study was obtained from the Ghana Health Service Ethics Review Board.

### Data analysis

All transcripts were reviewed by the research team. During this review, any personally identifiable information was removed to ensure that no sensitive information remained in the final transcript. The analysis utilised reflexive thematic analysis as described by Braun and Clarke (16). This method involves a six-step approach (17). The first step involves familiarisation with the data. The research team individually read the transcripts multiple times while listening to the recorded interviews to become familiar with the content before proceeding to the coding phase. During the second phase which is coding, both deductive and inductive coding approaches were employed to create the final codebook. This was done by reading through the data with the research questions in mind while also paying attention to any significant statements and concepts that resonated across the transcripts. These were labelled with colours. The initial themes were generated as the third phase when the research team grouped and regrouped similar codes to explore patterns of similar meanings. This was followed by the fourth and fifth phases where the entire research team convened to review the initial themes and develop, discuss, refine and name the themes, ensuring the reliability of the findings. The final phase was the writing of the report. This was done by including participants’ quotes and the analytical commentary of the researchers.

### Methodological trustworthiness

To ensure the integrity and rigour of this qualitative study, the researchers adhered to the established framework of trustworthiness proposed by Lincoln and Guba, and Ahmed (18, 19), focusing on four pillars: credibility, dependability, transferability, and confirmability.

Credibility was maintained through member checking, where participants from across the focus groups were invited to review the study's findings to ensure the interpretations accurately reflected the information they provided. Furthermore, researchers discussed their own biases, before data collection and during analysis to ensure that the data authentically represented the participants’ views.

By maintaining a transparent audit trail and proper documentation of every phase of the study and providing detailed description of all the step-by-step processes followed throughout the study including the justifications for methodological decisions, the data collection procedures, and analysis, dependability and transferability were ensured.

Confirmability was ensured by having the research team members independently engage with transcripts before coming together to agree on themes and write the final report. Thus, it was ensured that researchers’ own perspectives did not influence the participants’ narratives.

## Results

### Sociodemographic characteristics of participants

A total of 32 participants took part in this study. Majority of the participants (17) were midwives while 15 were nurses. Most of them (17) had a bachelor's degree, 11 had a master's degree, three held a diploma and one was a certificate holder. The differences in rank however does not influence the basic skills needed to provide SRH. Regarding gender, marital status and religion, the majority (31) were females with only one male. Twenty- nine were married with three being single. Thirty-one of the participants identified as Christians and one identified as a Muslim. Find details in Table 1 below.

**Table 1:** Sociodemographic characteristics of participants (*N* = 32).

Characteristics	Frequency	Percentage (%)
Age (years)		
25–30	1	3
31–35	5	16
36–40	13	41
41–45	8	25
46–50	3	9
51–55	2	6
Specialty		
Midwife	17	53
Nursing	15	47
Years of Practice		
1–5	1	3
6–10	7	21
11–15	12	38
16–20	6	19
21–25	5	16
26–30	1	3
Rank/Position		
Saff Nurse	2	6
Senior Staff Midwife	3	9
Nursing Officer	2	6
Midwifery Officer	6	19
Senior Midwifery Officer	3	9
Senior Nursing Officer	3	9
Principal Nursing Officer	7	21
Deputy Chief Nursing Officer	2	6
Chief Nursing Officer	2	6
Deputy Director of Nursing Service	2	6
Qualification		
Certificate	1	3
Diploma	3	9
BSc.	17	53
MPhil	3	9
Masters	8	25

### Study themes

The study explored the perspectives of nurses and midwives on male involvement in sexual and reproductive health (SRH) in Ghana. The thematic analysis of the focus group revealed several themes and sub-themes that highlight the benefits, barriers, importance, and challenges to male involvement in SRH. In all, three themes and 9 sub-themes were generated. These themes and sub-themes are presented in Table 2 below and further discussed with verbatim quotes to support them.

**Table 2:** Study themes.

Themes	Subthemes
Acknowledged Benefits of male involvement	• Improved health outcomes for women • Reduced complications and morbidity • Enhanced support system
Barriers to Male Involvement	• Societal, cultural and religious expectations • Difficulty in convincing male partners to participate
Enhancing male involvement	• Conceptualization of male involvement • Increased desire to encourage male involvement in SRH issues • Increased education and awarenessCapacity building

### Acknowledged benefits of male involvement

Participants acknowledged several benefits of involving male partners. They expressed how involving male partners in SRH is associated with several benefits based on their experience of working in SRH units. These benefits included improved health outcomes for women, reduced complications and morbidity, enhanced support systems and increased men's appreciation for reproductive health issues.

#### Improved health outcomes for women

Concerning the benefits of male involvement in SRH, all participants described how male involvement can contribute to improved health outcomes for women. They expressed the view that the presence of male partners during antenatal care and labour leads to better monitoring of the woman's health because the partner receives the same information provided to the woman and helps her to adhere to the recommendations for good health outcomes.

Some of the participants had this to say:

“When men accompany their partners to the clinic, they help ensure that the women take their medications and follow dietary recommendations made by the nurses or midwives.” (A2)

Another participant had this to say:

“ ….when the men listen to most of the education, they support the women. The husband/ partner will be a reminder, always reminding the woman. A client told me it was the husband who makes sure she does the right things because she brought him to the facility to listen to the education that was given…” (A1)

Participants underscored the benefits of male involvement in the health outcome of the women. The woman's nutrition and other health related issues are addressed easily when the partners are educated and encouraged alongside their partners. According to the nurses and midwives, this has a rippling effect on their work as compliance of their clients to nutrition and health advises leads to improved health outcomes.

#### Reduced complications and morbidity

Several of the participants indicated that an important benefit of male involvement is that it leads to improved maternal health outcomes, which translates into a reduction in complications and morbidity. According to them, when the men are involved in the care of their partners, they gain a better understanding of issues and act promptly when necessary to avoid delays.

“In my facility, the clients that usually have their partners involved in the pregnancy journey, we usually don't have problems with them. At a point, we realized that our clients were having recurrent UTI (urinary tract infection), we decided to involve their partners. Some (partners) were able to report and did the test and got treated with their partners we realized that our UTI reports rate went down.” (B4)

Another participant clarified how male involvement minimizes complications

“So, when they come, with sexually transmitted infection (STI), we need to involve the partners too, because partner treatment is very important. Because if you don't involve them, there'll be re-infection, and they'll come back again. So, we educate them to come with their partners. When you come, we ask you to bring your partner for him or her to be treated.” (B1)

Participants believed that involving males in the care of their partners provides a window for addressing complications for conditions that requires treatment for both. The males are enlightened with the right information, resulting in limited influence from culture and other societal practices.

#### Enhanced support system

Partner support was considered by the participants as very important in all aspects relating to health. Most of the participants emphasised that male involvement fosters an increased sense of appreciation and serves as opportunity for essential support system for women. Many of the participants indicated that male involvement is a positive concept that should be encouraged. Participants were of the view that some of the negative reactions or apathy displayed by some men towards SRH issues are due to a lack of information or awareness of the important role men play in SRH. Involving men and providing the right SRH information is key in increasing their appreciation. It provides emotional and psychological support among others. The participants made several statements to indicate how male involvement serves as support.

“We encourage male involvement because when men are involved, women feel at ease for example during labour or even any other reproductive health issue. They know they have someone to rely on for physical and emotional support.” (E1)

The participants were of the view that when the men get involved, they learn a lot more about SRH issues and can support the women better because they gain understanding. The nurses and midwives find ways of encouraging the males to get involved.

“So, we get our men involved by organizing the daddy's clinic. They come and learn a lot then they can support their partners”….“we need our men also, because the “game” was played by two. So, we don't have to leave everything on the woman, we equip the men then they are able to support their partners….” (A2)

The support male involvement offers were reiterated by other participants who noted that because in the Ghanaian society and culture men of then have the final say on almost every issue, it is important to get the men on the side of women as far as SRH is concerned:

If the male is aware of the things that you need as a woman when it comes to SRH, he will stand with the woman so that society does not dictate what she is supposed to do. When the male partner is well informed, he is more likely to support the woman …. especially emotionally. (C1)

With proper information through involvement, men will support their women to adhere to the recommendations of their healthcare providers against other community opinion.

### Barriers to male involvement

The involvement of men in SRH is increasingly recognised as essential for improving maternal health outcomes. However, despite the benefits articulated by participants in this study, they mentioned various barriers that hinder the participation of males in these critical health areas. The barriers identified by participants include, Social and cultural expectations, lack of education and awareness of SRH issues among men, and issues of trust and decision- making among partners.

#### Social, cultural and religious expectations

Majority of the participants identified societal and cultural expectations as significant barriers to male involvement. Almost all of them talked about traditional beliefs that designate reproductive health as a woman's responsibility. Cultural narratives often define men who follow their partners to the hospital as being overly attached. Yet the absence of the men prevents them from having firsthand information about the care being provided. The following are some of the narrations provided by participants:

“Men often see antenatal care as a ‘woman thing’ and feel that their presence is not necessary” (D6)

Others had this to say:

“We know in our Ghanian setup, when you are pregnant or you give birth, then it is your mother's duty or your mother-in-law's duty to come and support and all that. But we need our men also, ….So, they don't have to leave everything on the woman….” (A2)

“so, like I mentioned earlier, if the woman comes to the clinic alone and goes to the house and talks, maybe with the husband and the mother in-law, she may be seen as being demanding or disrespectful.” (B1)

Most of the examples participants gave concerning barriers were related to family planning and comprehensive abortion care of which partner consent was a major barrier.

“So, when we send them to the family planning unit, they are counselled properly with their husbands. Those of them who don't come with their husbands sometimes also feel that they need their husbands. Our community is a Muslim community. The women think that their husbands will leave them if they docertain methods” (B6)

“Some of the women, if I advise them on family planning, they say, I want to discuss it. But others will accept it without their husband knowing. Especially the Muslims, if they come after delivery and you discuss with them, they say, I want to discuss with my husband, if not, it's a sin against the Quran. Because if I agree, and he doesn't, what will I do? I'll not do anything. I'll just give birth.” (B1)

#### Difficulty in convincing male partners to be involved

The involvement of male partners in reproductive health is a vital aspect of promoting an optimal health outcome for both partners. However, this involvement is often hindered by deeply ingrained cultural and social beliefs that view reproductive health as a women-only concern. Participants indicated this mindset, perpetuated by social norms and expectations, deters many men from actively participating in antenatal care, family planning and other reproductive health related issues. Additionally, services provided at most SRH units are usually women-centred making men feel out of place within the space as reiterated by some participants.

“Some of the males feel oh, antenatal is a woman thing, family planning is a woman thing, I mean those things are women things because we are the receivers of those services more often, so they feel it is a woman thing we don't really need to be there” (E6)

We must erase that attitude.. that when the males are not involved or they are not present during the care, they are not going to believe the woman (C4)

### Enhancing male involvement

Promoting and sustaining male involvement in SRH has positive health outcomes for individuals, families and communities. However, it has not been fully embraced by all. As such, strategies for enhancing it should be explored and utilized. From the data, enhancing male involvement emerged as a theme with four sub-themes “conceptualization of male involvement”, increased desire to encourage male involvement in SRH matters “increased education and awareness” and “capacity building”.

#### Conceptualization of male involvement

How healthcare providers conceptualize male involvement is an important step in its promotion. Participants believed there was no clear policy on the modalities of involving males in SRH. According to them the extent to which males are involved depends on many factors and circumstances such as the nurse/midwives understanding of male involvement, the physical or structural set-up of the health facility and even the community and/or organizational culture. These are some of the perceptions participants had…

“When we talk about male involvement, to me, it means that the man must understand what the female reproductive process is, …. Because it takes the man and the woman to ensure or maintain a good reproductive health. If the man understands very well what the woman goes through, he will be able to support…. For me, man understanding the reproductive health process is very important.” (A1)

“So when we talk about male involvement, I don't think it necessarily mean the man following her to the clinic on every antenatal clinic or postnatal or during labour. Maybe the partner may not even be around, but you can still be involved in the care … that's why we take the partner's numbers. You can, call the partner and give him some information” (B3)

Interestingly majority of the participants acknowledged the complexity of determining the extent to which a male partner can be said to be involved in SRH matters beyond their physical presence at the facility. Based on how they conceptualize male involvement some participants found other means of engaging the male partners.

#### Increased desire to encourage male involvement in SRH issues

The participants expressed their desire and appreciation for involvement of males in SRH issues. Most of the participants indicated that male involvement is a positive concept that should be encouraged. They indicated their acceptance and appreciation by saying:

“Getting the males involved when it comes to SRH is very good because it has a whole lot of benefits to everyone to the males themselves and even to us nurse and midwives. It makes our work easy” (E1)

Emphasising how male involvement facilitates the work of the nurse/midwife, another participant had this to add:

“Male involvement in reproductive health is very important … it helps you to work smoothly and then the woman is also at peace, having a sound mind to go through whatever process she's going through” (A5)

Participants were of the view that some of the negative reactions or apathy displayed by some men towards SRH issues is due to lack of information or awareness of the important role men play in SRH. Involving men and providing the right SRH information is key in increasing their appreciation. This was expressed by a participant;

“There are times we take them on a tour to the theatre to see the instruments, the caesarean section videos, so that they'll appreciate what their wives go through just to bring forth a human being.” (C7)

#### Increased education and awareness

Although male involvement in SRH is not an entirely new initiative it is important that more education is provided, and awareness created to enhance its acceptance and its benefits. Participants identified more education and awareness as one of the sure strategies to enhance male involvement.

A participant had this to say:

“I think the male involvement for me, we shouldn't even wait for us to get a client and then we now try involving the partner of the client, but we should do community kind of durbar, we need to get the men more involved from all aspects so….. on social media, we have telecommunications where we educate them on SRH. I mean, we should give a general education on it.” (E2)

Participants stressed the need to adopt strategies to reach the men and educate them

“We also get the men on board. In fact, my former facility, we were not having the males. They were not coming. So, we decided to visit them in their homes. We took their addresses and planned home visits. And then we meet the man and the woman in the house then we talk about a lot of issues,… We realized that most of the men got involved.” (A1)

Similarly, a participant also emphasised the need for education on the subject.

“When men are educated about the reproductive process, they are more likely to engage positively in discussions about family planning.” (B2)

Another participant narrated what is done in her facility to enhance male involvement;

“… in my facility we do pregnancy school and we involve the males who want to always accompany their wives to the clinic. ….we discuss family planning…. even before delivery, as part of the pregnancy school we send them to the labour ward and orient them on whatever goes on there. …we have a page for pregnant women and their partners on WhatsApp so, the men can ask questions.” (B4)

#### Capacity building

Participants expressed the desire to receive some further training and education on the concept of male involvement to equip them to advocate for getting male partners involved in SRH matters. Most participants reported a lack of formal training in practical male engagement strategies, leaving them to navigate complex cultural and religious, and infrastructural barriers by just doing what they think is best under the circumstances at their individual facilities. This is how they expressed their desire:

“Okay. When we talk about training on male involvement. I haven't received any special training on male involvement, but I will say that I had the knowledge through school and then training on the job. So that is how I had a bit of knowledge about male involvement in sexual and reproductive or the concept of male involvement in that area.” (D5)

A similar point was made by another participant saying

“I'm trying to recollect if I've had a specific training when it comes to male involvement. And I can't recall because I feel when you look at service provision, you take the antenatal book, you take family planning, you take a lot of indicators when it comes to providing services, especially SRH. You have a component that is asking about male/ partner involvement. Probably that has informed us of getting our males involved.” (A2)

To maximise the benefits of male involvement, there is a clear institutional need for professional capacity building. Training programs must equip healthcare workers with standardized, professionally informed strategies to navigate the socio-cultural dynamics unique to their facilities, moving away from ad-hoc approaches towards evidence-based practice.

One participant had this to say….

“Concerning training or CPD on male involvement, I have not heard one before. or went for any training before. I am not surprised why some midwives think male involvement is not a whole big deal, so they handle male involvement anyhow. I think if there can be training in that aspect for health workers who are into SRH, it is really going to make this male involvement attractive to those of us that don't value it. I think it is really going to help.” (C6)

Another added …

“ I don't think there have been any official training for us on male involvement…, if maybe there can be a training strategically on how to go about all those things involved it will be good. We are saying we should involve the males so we should know how. I think there should be some training for health workers not only midwives but for all those who deal with reproductive and childcare, then it will be of help”. (B4)

## Discussion

The findings from this study underscore the multifaceted benefits of male involvement in SRH from the perspectives of nurses and midwives in Ghana. Although the participants were mostly females it must be acknowledged that females dominate the SRH space as far as the nursing and midwifery workforce is concerned yet they are the ones to promote male involvement. Participants’ views as expressed in this study were based on their professional experience rather than their own personal male support experiences. This notwithstanding, the gender dynamics in this study mirror the real-life situation where SRH services are predominantly rendered by females. This can potentially discourage some men from accompanying their partners into an all-female environment where they may feel out of place. Increasing male representation among professionals providing SRH services has been recommended as one of the strategies for improving male involvement (2). The participants highlighted that engaging male partners in antenatal care and other reproductive health services leads to improved health outcomes for women, reduced complications and morbidity, enhanced emotional support, and an appreciation for the significance of male involvement in SRH issues. These benefits align with existing literature that recognizes men's critical role in supporting women's health during pregnancy, childbirth, and beyond (8, 20).

The emphasis on improved health outcomes for women resulting from male attendance at health facilities resonates with other research indicating that male involvement can facilitate better adherence to medical advice and treatment plans (8). This is reflected in participants’ accounts of how men who accompany their partners to clinics are better informed about health instructions, leading to more effective health practices at home. For instance, as Participant A1 pointed out, men can act as reminders and advocates for their partners’ health needs. Such dynamics not only enhance health adherence but can also transform power relations within the household, fostering a partnership model as opposed to a traditionally patriarchal one that often marginalizes women's health decisions (21).

Furthermore, participants emphasized the importance of male involvement in reducing complications and morbidity associated with STIs and other reproductive health issues. Studies have demonstrated that neglecting male partners in STI management can lead to reinfection and complicate treatment protocols (20). Thus, encouraging men to support their partners and participate actively in their health care can significantly contribute to achieving better overall SRH outcomes.

The emotional and psychological support provided by male partners was another consistent theme among participants. This finding corroborates literature indicating that support from male partners during pregnancy and childbirth can mitigate maternal anxiety and enhance the overall well-being of women (12). The emotional backing described by participants, such as those during labour, shows how vital male presence is, not merely as a logistical support but as a source of comfort and reassurance.

Despite the myriad benefits identified, the study also revealed several barriers that hinder male involvement in SRH. Social, cultural, and religious expectations frequently dictate that reproductive health is solely a woman's domain. This sentiment mirrors findings from research in similar contexts, highlighting how cultural norms can restrict men's participation (3). Many men perceive antenatal care as a “woman's thing,” which complicates efforts to engage them in the healthcare process. Additionally, the influence of traditional beliefs in Ghanaian society often sidelines the male partner, relegating them to passive observers rather than active participants in their partners’ health journeys (22). Despite this general notion, it is worth noting that other studies in Ghana have argued that masculinity does not always resist caregiving roles and support for women's SRH needs. On the contrary, some male ideologies such as being a responsible provider, who takes care of his partner rather encourage some men to be involved in the healthcare needs of women. Additionally, intergenerational knowledge over the years is gradually and positively changing the notion of SRH matters being “a woman's thing” (23, 24).

Moreover, the fear that educational discussions on SRH may contradict established societal norms discourages both men and women from seeking care together. Participants pointed out that some women express reluctance to discuss family planning with their partners due to fears of retribution or misunderstanding, particularly in Muslim communities. This is concerning, as it points to a need for culturally sensitive approaches that respect existing beliefs while promoting healthier practices.

To bridge the gap identified in male involvement, there is a need for targeted interventions that promote awareness of the importance of male participation in SRH. Educational programs designed specifically for men can help them realize their pivotal roles in the process. The concept of “daddy's clinics,” as mentioned by participants, offers a tangible solution, providing a space where men can learn about reproductive health in a supportive environment, thereby equipping them with knowledge that enhances their partner's health outcomes (22).

The theme of enhancing male involvement in SRH, as expressed by participants, reflects a crucial aspect of modern health care that aims to improve outcomes for women and families. The data revealed three key sub-themes: conceptualization of male involvement, increased education and awareness, and capacity building. Each sub-theme highlights vital areas that must be addressed to effectively nurture male participation in SRH.

A primary finding from this study indicated that the conceptualization of male involvement in SRH is often ambiguous and lacks a clear framework. Participants noted the absence of explicit policies guiding male involvement in healthcare settings. This echoes observations from other researchers who have highlighted the need for better-defined roles for men within the context of reproductive health (25). The perception that male involvement encompasses understanding women's reproductive health processes is critical. Participants emphasized that knowledge could translate into support, thus fostering a partnership that promotes mutual understanding (26).

Moreover, the suggestion that male involvement does not solely mean attending clinics but can extend to receiving and acting upon information suggests a more inclusive conceptualization. This aligns with findings by Anbesu, et al. (27) who posit that male involvement can manifest in various forms, including home-based education and communication strategies. Most importantly, the role of healthcare practitioners in bridging the knowledge gap is pivotal, as they can facilitate the flow of vital information between partners, thereby enhancing cooperation in reproductive health matters (13).

The emphasis on increased education and awareness serves as a significant strategy to enhance male involvement. Participants unanimously agreed that targeted outreach and community engagement would be effective. This mirrors the suggestions made by Bagenda et al. (8), which advocate for community-based interventions to educate men about their roles in SRH. Innovative approaches such as social media campaigns, home visits, and specialized programs like pregnancy schools can effectively demystify SRH topics for men, making them more approachable.

Community engagement in Ghana has shown promise in influencing attitudes towards family planning and reproductive health (28). This further supports the notion that proactive engagement can desensitize the societal stigma around male involvement in SRH, making it a normalized aspect of reproductive care (29). As healthcare providers, nurses, and midwives have a pivotal role in initiating dialogue within communities—a strategy viewed as essential for enhancing male involvement in SRH services.

Capacity building emerged as a critical need among participants. The lack of formal training specifically targeting male involvement highlights a significant gap in the education of healthcare providers. This finding is consistent with previous research indicating that healthcare providers often feel insufficiently equipped to engage men in SRH (30). As articulated by participants, the desire for training transcends basic information; it encompasses the need for skills in communication and strategies for effective engagement with male partners.

Integrating training on male involvement into existing professional development for nurses and midwives could serve to enhance service delivery significantly. Studies have shown that targeted training can lead to more effective engagement practices and, ultimately, greater male participation in reproductive health services (31). By prioritizing male involvement in training curricula, health institutions can cultivate a workforce that is prepared to confront and reverse the traditional norms that have historically marginalized men's roles in reproductive health.

Generally, while studies in similar context (32) report benefits of male involvement in reproductive and maternal health, other studies (33) found limited influence on women's health outcomes. This difference presents the importance of context sensitivity to addressing issues on male involvement.

### Implications of the study

The study's findings indicate that nurses and midwives recognize the benefits and barriers associated with increasing male involvement in SRH and are generally willing to support this initiative. However, the findings showed that there is currently a lack of sufficient knowledge, empowerment and resources to actively promote male involvement. This suggests that, despite an awareness and acceptance of the importance of male participation, there is an absence of structured, proactive efforts to encourage it. Therefore, developing a comprehensive, policy-driven agenda aimed at promoting male involvement across all aspects of SRH is essential. Ghana Health Service and other relevant ministries could include clinic hours for males and community outreach programmes targeted at males. Pre-service and in-service training for nurses and midwives on male involvement by Ministry of Health could also empower health workers on context specific approaches to encouraging male involvement. Such a framework would serve as a strategic tool for guiding interventions, fostering active engagement, and enabling the systemic monitoring and evaluation of progress in enhancing male involvement in SRH overtime.

### Strength and limitations of the study

One of the key strengths of this study is its qualitative design, which allowed participants to freely express their perspectives, resulting in rich and in-depth data. Additionally, participants were recruited from various health facilities across different levels of care in Accra, providing a diverse range of insights while capturing common experiences.

A limitation of the study is that the dynamics of male support in SRH may be heavily influenced by the socio-cultural norms specific to the geographical setting studied. Since this study was conducted in the Greater Accra Region which is predominantly an urban setting the perspectives of nurses and midwives who have worked in this region may differ from the those in the rural setting. Consequently, the findings may not be universally generalizable. Also, the participants were predominantly females limiting the perspectives of male service providers. Male healthcare providers may have offered additional insights regarding gender dynamics, service delivery challenges and strategies for engaging men. However, since the recommendations are broad, they can still be adopted to similar or different contexts with appropriate cultural and situational considerations.

## Conclusions

Promoting male involvement in SRH can potentially lead to timely decision-making and supportive care. Nurses and midwives recognize gender dynamics and can help reduce barriers through education, outreach, and policy advocacy. Empowering healthcare providers is essential to increase male involvement. Future research should explore men's perspectives on involvement and their expectations of nurses and midwives.

## Data Availability

The raw data supporting the conclusions of this article will be made available by the authors, without undue reservation.
